# Supplementary material for: Overcoming Aging-Associated Poor Influenza Vaccine Responses with CpG 1018 Adjuvant
Source: Vaccines (Basel). 2022 Nov 10;10(11):1894. doi: 10.3390/vaccines10111894 (PMC9695697; doi:10.3390/vaccines10111894)
Supplement: Supplementary file 1 [file vaccines-10-01894-s001.zip › vaccines-2019641-supplementary.pdf]

Supplementary Information

Overcoming aging-associated poor influenza vaccine responses with CpG 1018 adjuvant

Kang et al.

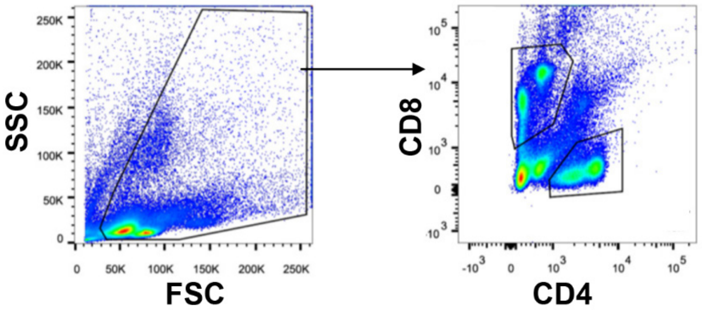

Figure S1. Gating strategies in Figure 5

Cells were first gated based on SSC and FSC and then based on CD4 and CD8 expression. Single positive (CD4+ or CD8+) T cells were gated for analysis of cytokine expression patterns.
